# Efficacy and Safety of Stroke Volume Variation-Guided Fluid Therapy for Reducing Blood Loss and Transfusion Requirements During Radical Cystectomy: A Randomized Clinical Trial

**DOI:** 10.1097/MD.0000000000003685

**Published:** 2016-05-13

**Authors:** Yu-Gyeong Kong, Ji Yoon Kim, Jihion Yu, Jinwook Lim, Jai-Hyun Hwang, Young-Kug Kim

**Affiliations:** From the Department of Anesthesiology and Pain Medicine, Asan Medical Center, University of Ulsan College of Medicine, Seoul, Republic of Korea.

## Abstract

Radical cystectomy, which is performed to treat muscle-invasive bladder tumors, is among the most difficult urological surgical procedures and puts patients at risk of intraoperative blood loss and transfusion. Fluid management via stroke volume variation (SVV) is associated with reduced intraoperative blood loss. Therefore, we evaluated the efficacy and safety of SVV-guided fluid therapy for reducing blood loss and transfusion requirements in patients undergoing radical cystectomy.

This study included 48 patients who underwent radical cystectomy, and these patients were randomly allocated to the control group and maintained at <10% SVV (n = 24) or allocated to the trial group and maintained at 10% to 20% SVV (n = 24). The primary endpoints were comparisons of the amounts of intraoperative blood loss and transfused red blood cells (RBCs) between the control and trial groups during radical cystectomy. Intraoperative blood loss was evaluated through the estimated blood loss and estimated red cell mass loss. The secondary endpoints were comparisons of the postoperative outcomes between groups.

A total of 46 patients were included in the final analysis: 23 patients in the control group and 23 patients in the trial group. The SVV values in the trial group were significantly higher than in the control group. Estimated blood loss, estimated red cell mass loss, and RBC transfusion requirements in the trial group were significantly lower than in the control group (734.3 ± 321.5 mL vs 1096.5 ± 623.9 mL, *P* = 0.019; 274.1 ± 207.8 mL vs 553.1 ± 298.7 mL, *P* <0.001; 0.5 ± 0.8 units vs 1.9 ± 2.2 units, *P* = 0.005). There were no significant differences in postoperative outcomes between the two groups.

SVV-guided fluid therapy (SVV maintained at 10%–20%) can reduce blood loss and transfusion requirements in patients undergoing radical cystectomy without resulting in adverse outcomes. These findings provide useful information for optimal fluid management during radical cystectomy.

## INTRODUCTION

Bladder cancer is one of the most common cancers of the genitourinary tract in adults, and its incidence distinctly increases with age.^1,2^ In almost two-thirds of cases, the disease is superficial at presentation and involves the mucosal and submucosal layers or the lamina propria of the bladder, whereas ∼20% to 30% of patients have muscle-invasive tumors. Superficial bladder cancer is treated by transurethral endoscopic resection, which can be followed by endovesical therapy for patients at risk of disease recurrence and progression.^3^ In contrast, muscle-invasive bladder cancer is generally treated by radical cystectomy with pelvic lymph node dissection, which demonstrates 10-year recurrence-free survival rates of 50% to 59% and overall survival rates of ∼45%.^4,5^

Radical cystectomy combined with urinary diversion can result in substantial intraoperative blood loss and significant postoperative complications, which are reportedly in the range of 24% to 64%.^5–8^ In particular, intraoperative blood loss and blood transfusion are known to be associated with adverse postoperative outcomes in many clinical studies.^9,10^ Therefore, it is very important to establish surgical and anesthetic protocols aimed at minimizing intraoperative blood loss and subsequent blood transfusion requirements in order to improve postoperative outcomes. However, there is limited information about the optimal fluid management that can decrease intraoperative blood loss and blood transfusion during radical cystectomy, which are associated with complex, long surgical procedures.

Stroke volume variation (SVV) is attracting increasing attention as a reliable index for protocolized fluid management (i.e., maintaining SVV at 10%–20%) to minimize vascular congestion in surgical areas and intraoperative blood loss during living-donor hepatectomy.^11,12^ Thus, there is the potential to apply SVV-guided fluid management to patients undergoing radical cystectomy who are at risk of bleeding and blood transfusion during surgery. With these considerations, we aimed to evaluate the efficacy and safety of SVV-guided fluid therapy to decrease blood loss and transfusion requirements during radical cystectomy. To this end, we compared the amounts of intraoperative blood loss and transfused red blood cells (RBCs) between a control group (maintained at <10% SVV) and a trial group (maintained at 10%–20% SVV) during radical cystectomy. We also compared postoperative outcomes between these two groups.

## METHODS

### Patients

This study was a randomized clinical trial of 48 patients who underwent radical cystectomy at Asan Medical Center, Seoul, Republic of Korea between March and November 2015. The study protocol was approved by the Asan Medical Center Institutional Review Board (approval number: 2015-0147), and written informed consent was obtained from all of the patients. This study is registered on the international clinical trials registry platform (ClinicalTrials.gov: NCT02373735). The following groups of patients were excluded from our present analyses: (1) patients <20 years or >80 years of age; (2) patients who had cardiac arrhythmias; (3) patients who experienced significant heart failure or renal failure; (4) patients with a history of prior abdominal operations; (5) patients who refused to participate in this study; and (6) patients who required an emergency operation. In addition, in accordance with our institutional guidelines, patients who were required to use antiplatelet or anticoagulation medications such as plavix or aspirin discontinued these medications 7 days before radical cystectomy. We enrolled 48 patients scheduled for elective radical cystectomy and allocated these patients to two groups (control and trial groups). In accordance with the approved study protocol, 1 investigator created computer-generated randomization codes and enrolled the participants. This investigator assigned these subjects into the control or trial groups based on these codes, which were kept in sequentially numbered opaque envelopes. After anesthetic induction, these envelopes were opened by another investigator, an anesthesiologist assigned to conduct fluid management during the radical cystectomy procedures. An additional third investigator measured the primary and secondary outcomes in a blind manner. The surgeons were also blind to the group allocation.

### Anesthesia method

The single investigator controlled the ventilator settings and fluid management during the radical cystectomy procedure in all 46 patients. Anesthesia was induced using thiopental, rocuronium, and sevoflurane, and maintained using 2 vol% sevoflurane, a 50% oxygen/air mixture, fentanyl, and rocuronium. Mechanical ventilation was performed with a constant tidal volume of 8 mL/kg and a respiratory rate of 10 to 14 cycles/min without positive end-expiratory pressure. The end-tidal carbon dioxide tension was maintained between 35 and 40 mm Hg during radical cystectomy. Continuous electrocardiography, heart rate, body temperature, and peripheral oxygen saturation were routinely monitored. In addition, arterial blood pressure, stroke volume, SVV, and systemic vascular resistance were also monitored by inserting a 20-gauge catheter into the radial artery and connecting it to a Vigileo device (Vigileo/FloTrac; Edwards Lifesciences, Irvine, CA). A 3-lumen central venous catheter was also inserted into the right internal jugular vein to monitor central venous pressure (CVP). Intraoperative hemodynamic variables were collected at 5 time points (15 minutes after skin incision, 1 hour after skin incision, immediately after cystectomy, 1 hour after cystectomy, and at the end of surgery).

### Intraoperative Fluid Management Protocol

Fluid management followed a strict protocol in both groups (Table 1). Crystalloid (Hartmann's solution) were used as the maintenance administration. Two hundred milliliters of colloid (Volulyte^®^, 6% hydroxyethyl starch; Fresenius Kabi Deutschland GmbH, Germany) or 400 mL of crystalloid were used for bolus administration. The control group was infused with 6 to 10 mL/kg/h crystalloid until the end of the operation in order to maintain SVV at <10%. When the SVV value was ≥10%, maintenance administration was maintained at an infusion rate of 10 mL/kg/h crystalloid, and a bolus such as 200 mL colloid or 400 mL crystalloid was also administered (Table 1). In the trial group of patients, in order to maintain SVV at 10% to 20%, crystalloid were administered at an infusion rate of 3 to 4 mL/kg/h until the bladder was removed, followed by 6 to 10 mL/kg/h crystalloid until the end of the surgery. If the SVV value was >20%, the infusion rate was maintained at 10 mL/kg/h, and a bolus such as 200 mL colloid or 400 mL crystalloid was also administered for volume replacement. If the SVV value was <10% during the operation, the infusion rate of the crystalloid was decreased to 2 mL/kg/h and 0.5 g/kg mannitol was also administered to maintain SVV at 10% to 20% in the trial group (Table 1).

**TABLE 1:**
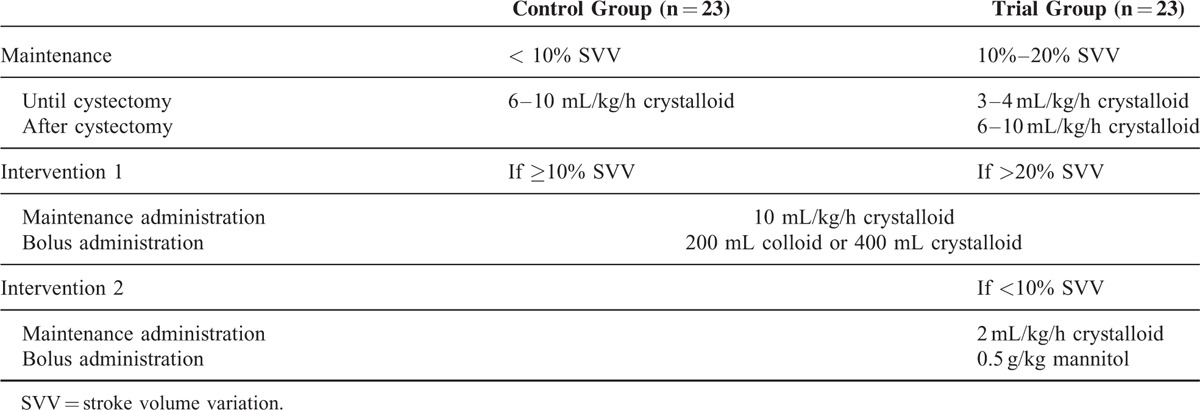
Intraoperative Fluid Management Protocol used for the Radical Cystectomy Patients in this Study

### Primary and Secondary Endpoints

The primary endpoints included the amounts of intraoperative blood loss and transfused RBCs between the control group (maintained at <10% of SVV) and trial group (maintained at 10%–20% of SVV) during radical cystectomy. Intraoperative blood loss was evaluated using both the estimated blood loss and estimated red cell mass loss. The estimated blood loss was measured by the sum of the volume of blood contained in suction systems and sponges. The estimated red cell mass loss was derived from the perioperative changes in the hematocrit and transfused RBCs using the following equation: red cell mass loss (mL) = patient's estimated blood volume (mL) × (preoperative hematocrit in % – postoperative hematocrit in %) + (transfused RBC in units × 213 mL × 70%). The estimated blood volume is 75 mL/kg for men or 65 mL/kg for women, the average RBC volume is 213 mL, and the average hematocrit for RBCs is 70%.^13,14^ During the radical cystectomy, the hemoglobin concentration was maintained at ≥8.0 g/dL and the transfusion of packed red blood cells was considered if it fell below this level.

The secondary endpoints were comparisons of the postoperative outcomes between the control and trial groups. All surgical and medical complications that occurred within 30 days after radical cystectomy were recorded. Records from the general ward and intensive care units, which were available in computerized databases, were analyzed. The serum lactate and base excess levels were measured on postoperative days 0, 1, and 2. Acute kidney injury was defined according to the classifications of Kidney Disease: Improving Global Outcomes (KDIGO).^15^ Time to first flatus and the initiation of a liquid diet were also determined after surgery. Gastrointestinal complications included peritonitis, gastrointestinal bleeding, anastomotic leakage, ileus, constipation, and gastrointestinal ulcers. Peritonitis, gastrointestinal bleeding, and anastomotic leakage were considered as complications if surgery was required.^16^ Ileus was defined as no evidence of bowel function with abdominal distension requiring the cessation of an oral diet by postoperative day 5, and constipation was diagnosed if there was no passage of a stool without signs of ileus by postoperative day 5. Gastrointestinal ulcers were diagnosed on gastroscopy.^17^ A postoperative pulmonary complication was defined as the occurrence of at least 1 event on the following list: respiratory failure (postoperative partial pressure of oxygen [PaO_2_] <60 mm Hg while breathing room air, ratio of PaO_2_ to inspired oxygen fraction <300, or arterial oxyhemoglobin saturation measured with pulse oximetry <90%, and requiring oxygen therapy), pleural effusion (diagnosed on chest radiography), atelectasis (diagnosed on chest radiography), pulmonary edema (diagnosed on chest radiography), and pneumonia (leukocytosis, body temperature >38°C, clinical signs of pneumonia, and requiring antibiotics).^18^ Cardiovascular complications included myocardial infarction (troponin T >0.05 μg/L and either ST-T segment changes or new Q-wave changes), congestive heart failure (diagnosed on chest radiography and requiring diuretics), and cardiac arrhythmia (diagnosed by electrocardiography and requiring new medications or electroconversion).^16^ Infectious complications included urinary tract infection (body temperature >38°C in the previous 24 hours, leukocytosis, bacterial counts >100, 000 in urinary analysis, and antibiotics required), sepsis (bacterial infection and at least 2 of the following list: tachypnea, tachycardia, hyper- or hypothermia, leukocytosis or leukocytopenia, or positive blood culture), wound infection (requiring a secondary operation), and intraabdominal infection (requiring drainage or antibiotics).^17^

Ephedrine, phenylephrine, or norepinephrine was administered to patients in both groups if hypotension was observed (i.e., mean arterial blood pressure [MAP] <65 mm Hg). In addition, hemodynamic measurements were performed when the hemodynamic variables were stably maintained at 5 to 10 minutes after vasopressor administration. The duration of hospital stay was determined from the day after radical cystectomy, and the intensive care unit admission rate was calculated as the number of patients who were admitted to the intensive care unit after radical cystectomy.

### Statistical Analysis

In our previous pilot study, the mean intraoperative blood loss measured by the sum of the volume of blood contained in suction systems and sponges were 1200 ± 480 mL in the control group and 850 ± 280 mL in the trial group. We assumed that SVV-guided fluid therapy would reduce the 30% rate of intraoperative blood loss. Accordingly, a sample size of 21 patients in each group was required, with a type I error of 0.05 (2-tailed) and a power of 0.80. Therefore, 24 patients per group were enrolled to compensate for possible dropouts. Categorical data are expressed as number (percentage) and were analyzed using the chi-square test or Fisher exact test, as appropriate. Continuous data including demographic characteristics, preoperative laboratory variables, and intraoperative variables except hemodynamic parameters are presented as mean ± SD or median (interquartile range) and were analyzed using *t* test or Mann–Whitney *U* test, as appropriate. In addition, the intraoperative hemodynamic variables and postoperative lactate and base excess values are expressed as least square mean (95% confidence interval) and were compared using linear mixed model. Bonferroni correction was employed in cases of multiple comparisons. A *P* value <0.05/n (n = number of comparisons) was considered statistically significant when multiple comparisons were conducted, and a *P* value <0.05 was considered statistically significant in other cases. All statistical analyses were performed using SPSS for Windows (version 21.0; IBM-SPSS Inc., Armonk, NY) and Stata software version 13.1 (StataCorp LP, College Station, TX).

## RESULTS

**FIGURE 1:**
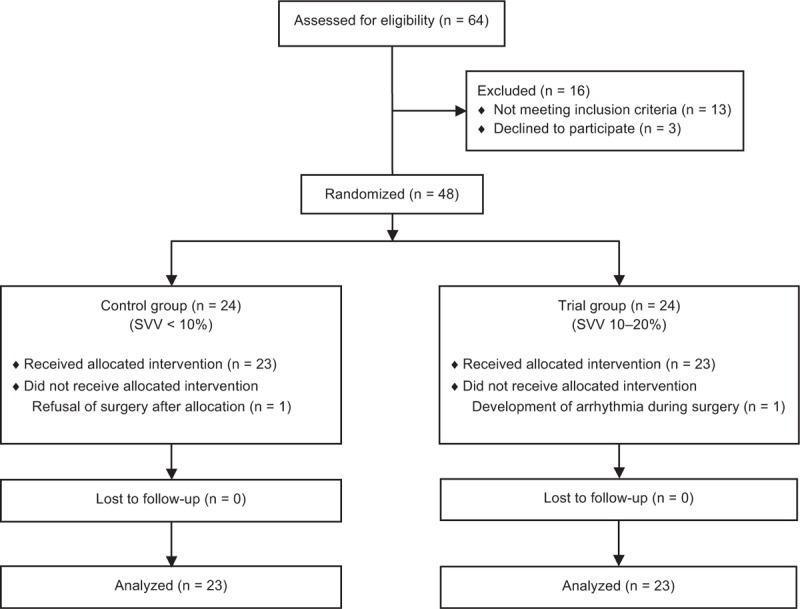
CONSORT flow diagram. SVV = stroke volume variation.

Of the 64 patients who underwent radical cystectomy in our initial study population, 16 cases were excluded (13 for failing to meet the inclusion criteria and 3 who refused to participate). Forty-eight patients were therefore enrolled in the present study cohort. After randomization, 1 patient in the control group did not receive the allocated intervention because of refusal of surgery after allocation and 1 patient in the trial group did not receive the allocated intervention because of the development of arrhythmia during surgery. These two patients were thus excluded from further analysis. Hence, 46 patients were included in the final analysis: 23 patients in the control group and 23 patients in the trial group (Figure 1).

There were no significant differences in the demographic characteristics or preoperative laboratory variables between the groups (Table 2). Table 3 provides a comparison of the intraoperative variables in the two study groups. There were also no significant differences in the anesthesia or operative times between the control and trial groups, or in the time from the induction of anesthesia to bladder removal between these groups [155 (118–198) minutes vs 134 (115–195) minutes; *P* = 0.678].

**TABLE 2:**
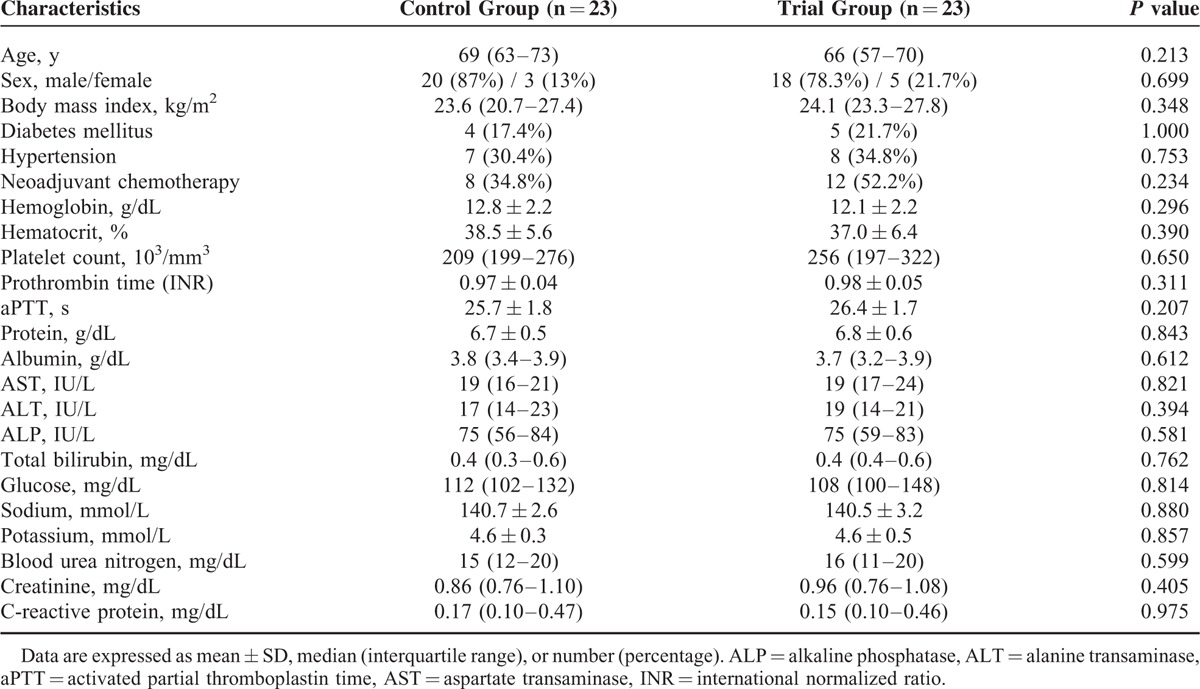
Demographic Characteristics of the Study Patients and Preoperative Laboratory Variables

**TABLE 3:**
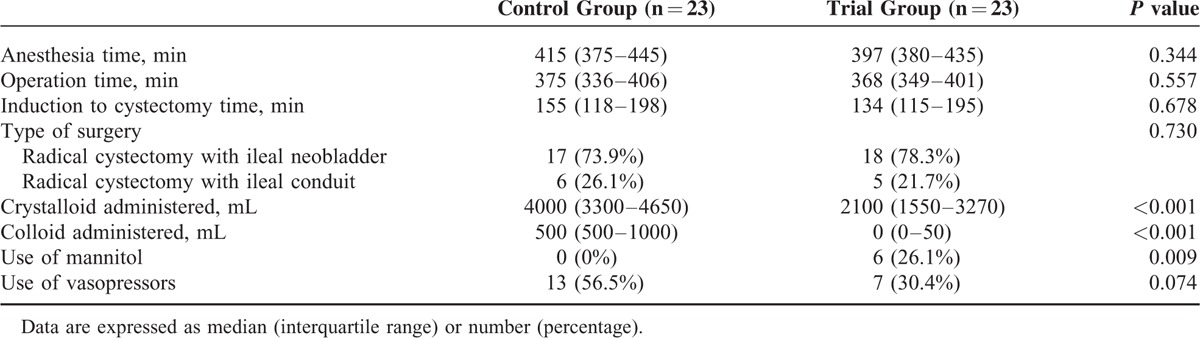
Intraoperative Variables

**FIGURE 2:**
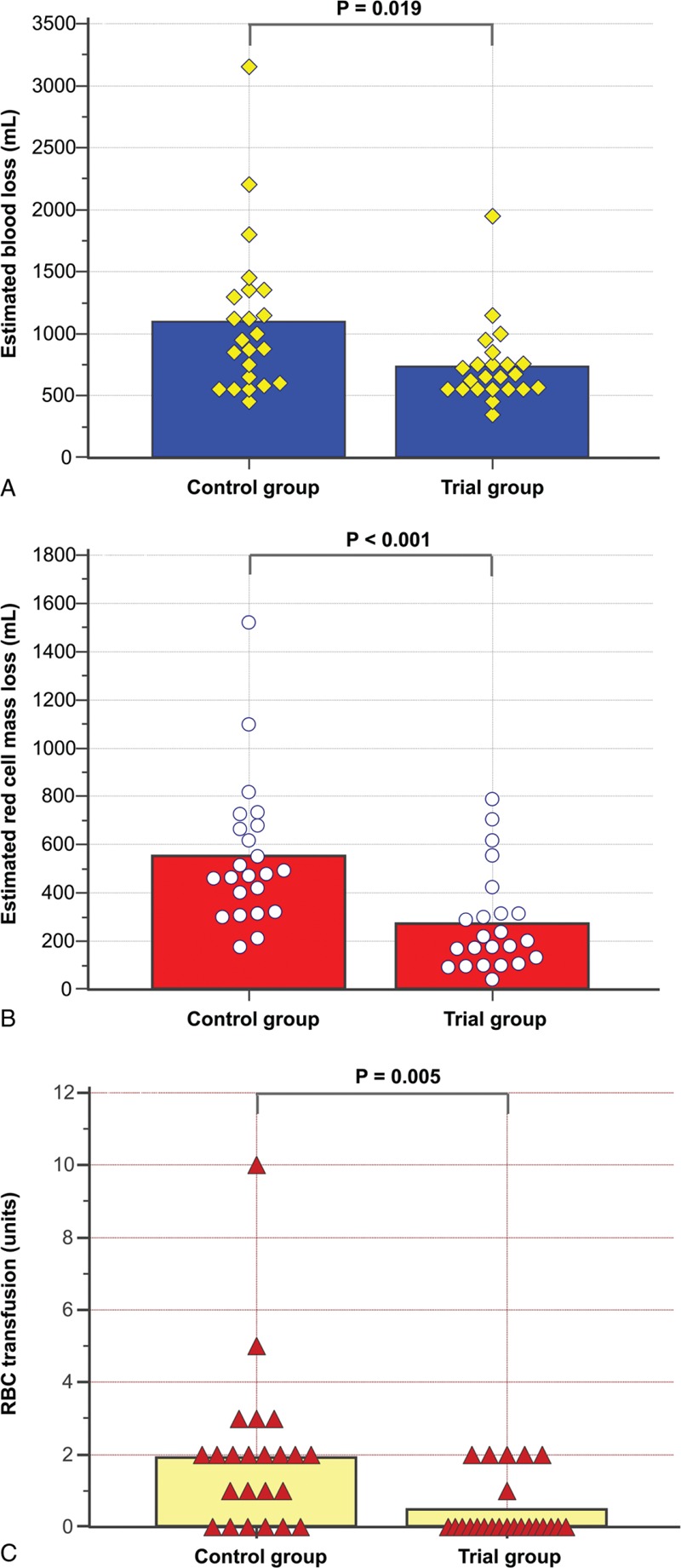
Comparisons of the estimated blood loss (A), estimated red cell mass loss (B), and transfused RBC (C) levels during radical cystectomy between a control group (maintained at a <10% stroke volume variation) and a trial group (maintained at a 10%–20% stroke volume variation). The estimated blood loss, estimated red cell mass loss, and RBC transfusion values in the trial group were significantly lower than those in the control group. The bars represent the mean values. The diamonds, circles, and triangles represent individual patients.

Figure 2 shows comparisons of the estimated blood loss, estimated red cell mass loss and transfused RBC levels between the control and trial groups. The mean hemoglobin and hematocrit levels before RBC transfusion were 7.35 ± 0.49 g/dL and 21.6 ± 1.5 %, respectively (7.30 ± 0.56 g/dL, 21.4 ± 1.7 % in the control group and 7.46 ± 0.35 g/dL and 22.0 ± 1.0 % in the trial group). The estimated blood loss, estimated red cell mass loss, and RBC transfusion requirements in the trial group were significantly lower than in the control group (734.3 ± 321.5 mL vs 1096.5 ± 623.9 mL, *P* = 0.019; 274.1 ± 207.8 mL vs 553.1 ± 298.7 mL, *P* <0.001; 0.5 ± 0.8 units vs 1.9 ± 2.2 units, *P* = 0.005) (Figure 2). The total amounts of crystalloid and colloid that were intraoperatively administered to the trial group were also significantly less than in the control group [2100 (1550–3270) mL vs 4000 (3300–4650) mL and 0 (0–50) mL vs 500 (500–1000) mL, respectively; both *P* <0.001] (Table 3). Mannitol was not administered in the control group, but was given to 6 patients (26.1%) in the trial group.

Table 4 lists the intraoperative hemodynamic variables during a radical cystectomy. SVV values in the trial group were significantly higher than in the control group during surgery. In addition, the CVP values in the trial group were significantly lower than in the control group from 1 hour after cystectomy to the end of surgery. However, there were no significant differences in stroke volume, systemic vascular resistance, or MAP between the two groups during surgery.

**TABLE 4:**
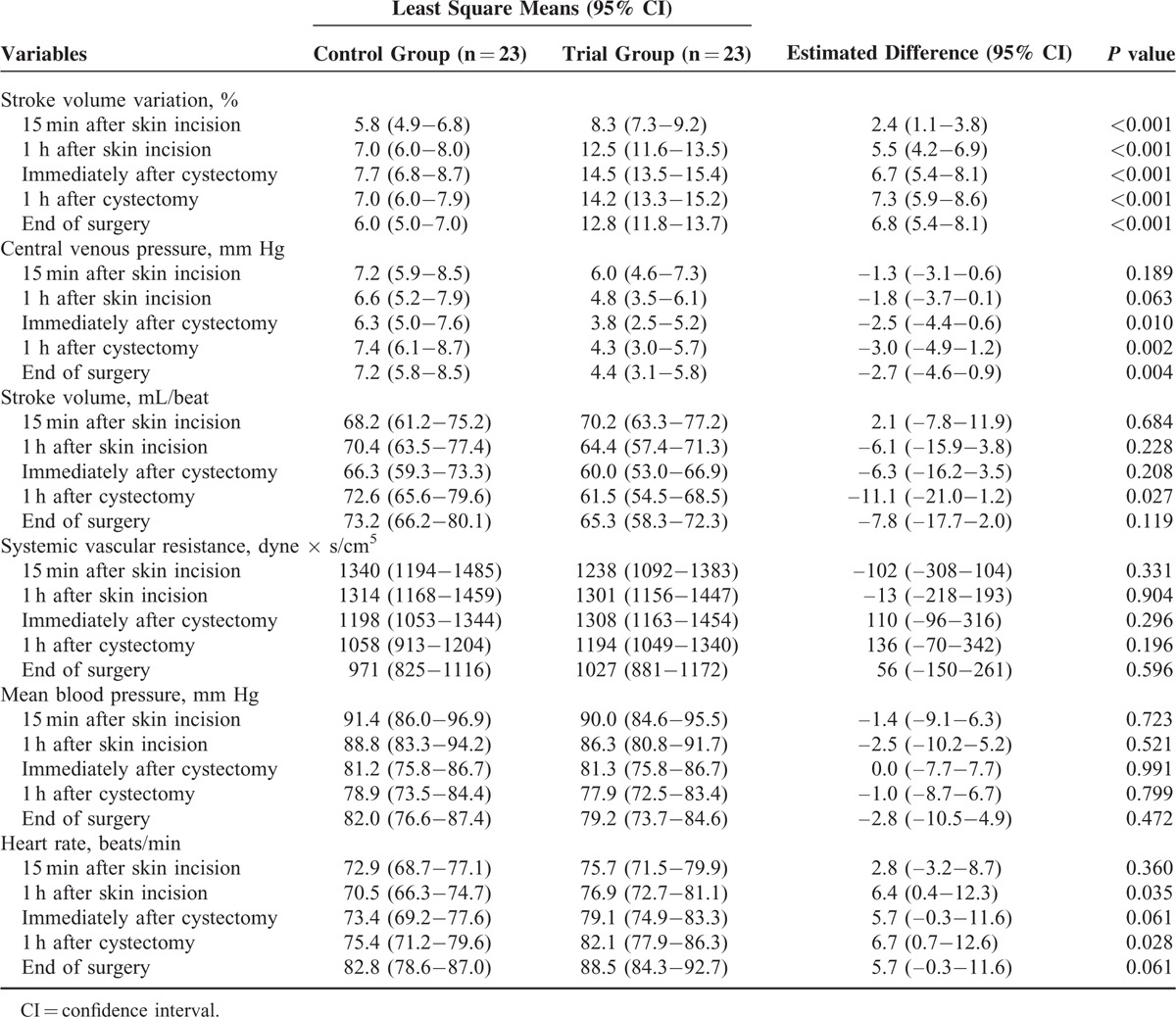
Intraoperative Hemodynamic Variables

There were no significant differences in the serum lactate and base excess levels on postoperative days 0, 1, and 2 between the two groups (Table 5). The incidence of acute kidney injury also did not significantly differ between the two groups [6 patients (26.1%) vs 8 patients (34.8%), *P* = 0.522]. The postoperative outcomes are presented in Table 6. There were no significant differences between the two groups in terms of time to first flatus or time to the initiation of a liquid diet after surgery. The most common complications observed were gastrointestinal complications, which occurred in 16 of 23 patients (69.6%) in the control group and 13 of 23 patients (56.5%) in the trial group. The majority of gastrointestinal complications were ileus (5 patients in the trial group vs 8 patients in the control group; *P* = 0.326) and constipation (8 patients in the trial group vs 7 patients in the control group; *P* = 0.753). Pulmonary complications were observed in 16 of 23 patients (69.6%) in the control group and 11 of 23 patients (47.8%) in the trial group; however, this was not significant (*P* = 0.134). There were also no significant differences in the duration of postoperative hospital stay or the intensive care unit admission rate between the two groups.

**TABLE 5:**
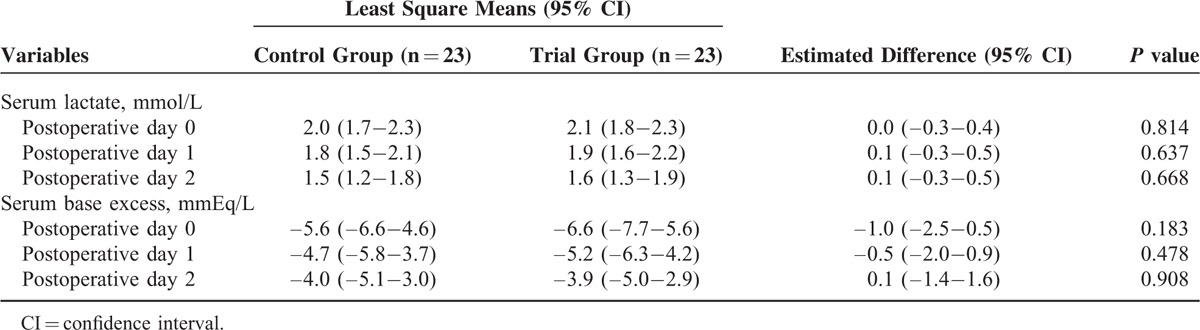
Postoperative Lactate and Base Excess Values

**TABLE 6:**
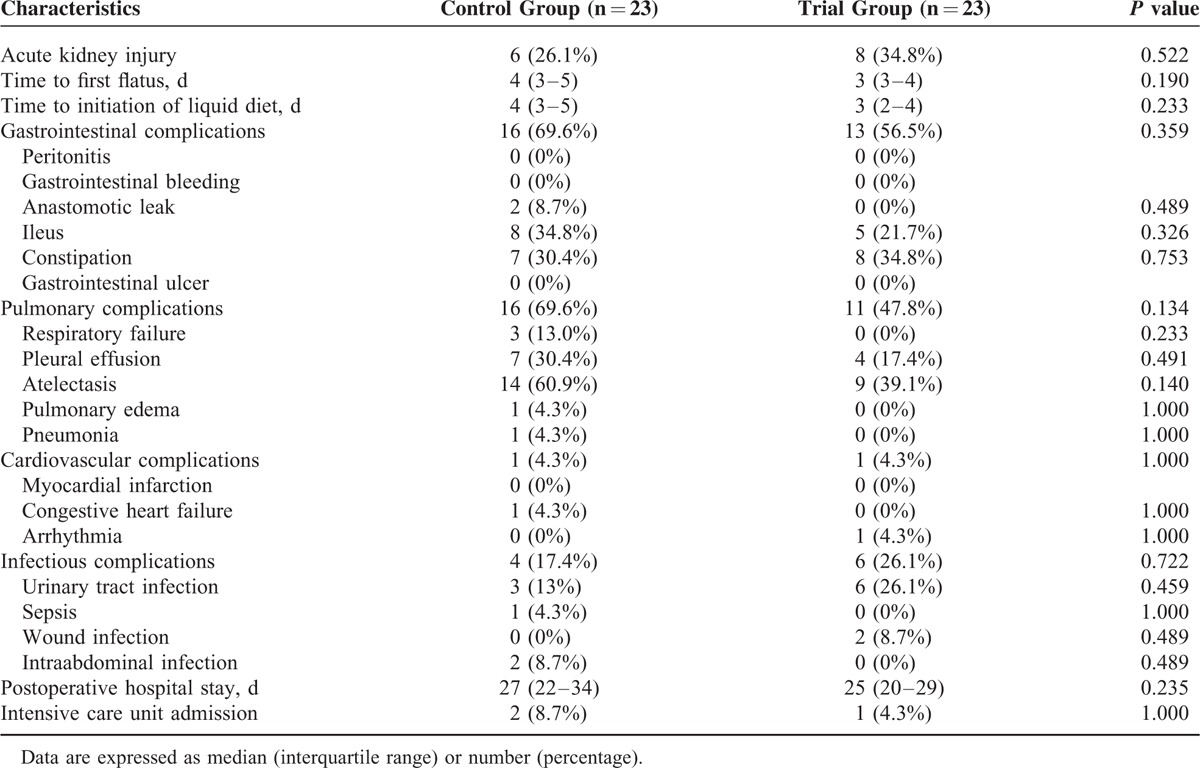
Postoperative Outcomes

## DISCUSSION

Our present study findings demonstrate that SVV-guided fluid management (maintaining SVV at 10%–20%) results in significantly reduced fluid administration, intraoperative blood loss, and RBC transfusion during radical cystectomy. In addition, we found no significant differences in the postoperative outcomes between our control group (maintaining SVV at <10%) and trial group (maintaining SVV at 10%–20%).

SVV, as a percentage change of stroke volume during the ventilatory cycle, is calculated from its relationship with the maximum stroke volume, minimum stroke volume, and mean stroke volume over a 20-second time interval and expressed as follows: SVV (%) = (maximum stroke volume – minimum stroke volume)/mean stroke volume.^19^ SVV is a useful dynamic variable that indicates fluid responsiveness in mechanically ventilated patients.^20^ However, the SVV is not applicable to patients with cardiac arrhythmias and spontaneous breathing because of its inaccurate interpretation of the respiratory variations in the stroke volume in these cases.^21^ As a more accurate measure, the SVV is recommended for use in patients in a mechanical ventilation setting and with a tidal volume of more than 8 mL/kg.^22,23^ Although SVV values may be increased by increasing the tidal volume,^24^ the SVV cut-off values for evaluating fluid responsiveness can be constant within a tidal volume range of 8 to 10 mL/kg.^23^ The optimal SVV cut-off value for evaluating fluid responsiveness was reported to be 10% in liver transplant recipients under mechanical ventilation with a tidal volume of 8 to 10 mL/kg.^19^ As a routine fluid management strategy for radical cystectomy based on our institutional protocol, maintaining the SVV at 10% to 20% has been observed to be more effective in reducing intraoperative blood loss and blood transfusion. To confirm this suggestion, we compared the intraoperative blood loss and transfused RBC level between our present study control group (maintained at a <10% SVV) and trial group (maintained at a 10%–20% SVV) under mechanical ventilation with a tidal volume of 8 mL/kg.

Radical cystectomy has been performed to treat muscle-invasive bladder tumors. However, because a radical cystectomy involves simultaneous surgery on the urinary tract, intestines, and lymph nodes,^25^ it is among the most difficult urological surgical procedures.^8^ Therefore, improvements in surgical technique, anesthetic management, and perioperative care have been attempted to reduce perioperative morbidity and mortality. However, despite recent technical improvements, a radical cystectomy is still associated with genitourinary, gastrointestinal, infectious, wound, cardiac, pulmonary, thromboembolic, and neurological complications, as well as intraoperative bleeding and subsequent transfusion.^26,27^ Importantly, blood loss during a radical cystectomy frequently occurs when dealing with the bladder vasculature and pedicles.^28^ Although intraoperative blood loss is common during this surgery, predicting its extent and the subsequent RBC transfusion requirements remains difficult.^28^ Furthermore, intraoperative fluid management during a radical cystectomy is widely variable.^25,29,30^ To the best of our knowledge, our present study is the first to evaluate the impact of SVV-guided fluid therapy on intraoperative blood loss and RBC transfusion requirements in bladder cancer patients undergoing a radical cystectomy.

Excessive intraoperative fluid administration is known to be associated with mortality and morbidity following major surgery.^31^ In contrast, restrictive intraoperative fluid management can reduce postoperative morbidity and shorten the hospital stay in patients undergoing elective intraabdominal surgery.^16^ In addition, intraoperative blood loss and blood transfusion are associated with postoperative adverse outcomes^9,10,32^ and high total hospital costs for radical cystectomy.^33^ Therefore, it is vital to prevent excessive intraoperative fluid administration and reduce intraoperative blood loss and subsequent blood transfusion requirements following this procedure to improve postoperative outcomes.

We have found that maintaining the SVV at 10% to 20% can markedly reduce the intraoperative fluid administration, blood loss, and RBC transfusion requirements during a radical cystectomy. The SVV is known to be a reliable dynamic index for evaluating fluid responsiveness.^34,35^ In our present study, the CVP in our trial group were lower than in our control group. It is thus possible that a lower CVP may predict a reduced intravascular filling state in a patient undergoing radical cystectomy, which could lead to decreased bleeding in surgical field. Therefore, maintaining the SVV at 10% to 20% as a target during fluid administration with a lower CVP could reduce intraoperative blood loss and lessen the RBC transfusion requirements during a radical cystectomy.

The serum lactate level is known to be an indirect but sensitive measurement of organ perfusion.^36^ In addition, the serum lactate level is correlated with the adequacy of intravascular volume.^36,37^ In our present study, the postoperative serum lactate levels did not differ between the control and trial groups. However, a normal lactate level does not signify that there is an adequacy between oxygen delivery and consumption.^38,39^ Therefore, the result regarding the effect of SVV-guide fluid therapy on an adequate balance between oxygen delivery and consumption during radical cystectomy needs to be interpreted with caution.

Our present results indicate that SVV-guided fluid management during a radical cystectomy may be not deleterious to renal function, as estimated by evaluating acute kidney injury defined by the KDIGO classification system. Acute kidney injury is an independent predictor of chronic kidney disease, along with age and the preoperative glomerular filtration rate, in radical cystectomy patients.^40^ In our present study series, the incidence of acute kidney injury did not significantly differ between the control and trial groups [6 (26.1%) vs 8 (34.8%), *P* = 0.522]. Since the calculation of sample size was based on primary endpoints, we could not ensure sufficient statistical power to compare the incidence of acute kidney injury between these study groups. Hence, further large studies will be needed to evaluate the influence of SVV-guided fluid therapy on acute kidney injury in patients undergoing radical cystectomy.

There were several possible limitations of note in our present study. First, surgical techniques can be associated with intraoperative blood loss and transfusion requirements. However, >1000 cystectomies have been performed at our center to date. Because our surgical team has extensive experience performing a cystectomy, the effect of the surgical techniques in this procedure on blood loss and transfusion requirement should have been minimal. Second, hemodynamic parameters such as SVV, stroke volume, and systemic vascular resistance were not used to evaluate postoperative outcomes. Therefore, our findings regarding the relationship between SVV-guide fluid therapy and postoperative hemodynamic changes need to be interpreted with caution. Third, maintaining the SVV at 10% to 20% may not be appropriate for a specific population such as septic patients.

In conclusion, SVV-guided therapy (maintaining SVV at 10%–20%) can reduce intraoperative blood loss and RBC transfusion requirements in patients undergoing a radical cystectomy and does not result in adverse outcomes. This indicates the potential of using SVV-guided therapy to avoid unnecessary and harmful volume overloading and decreases perioperative complications in major intra-abdominal surgeries.
