# Efficacy and Safety of Stroke Volume Variation-Guided Fluid Therapy for Reducing Blood Loss and Transfusion Requirements During Radical Cystectomy: A Randomized Clinical Trial: Erratum

**DOI:** 10.1097/MD.0000000000011129

**Published:** 2018-06-18

In the article, “Efficacy and Safety of Stroke Volume Variation-Guided Fluid Therapy for Reducing Blood Loss and Transfusion Requirements During Radical Cystectomy: A Randomized Clinical Trial”,^[1]^ which appeared in Volume 95, Issue 19 of *Medicine,* minus signs were left off of some numbers in Tables 4 and 5. The corrected tables appear below.

**Table 4:**
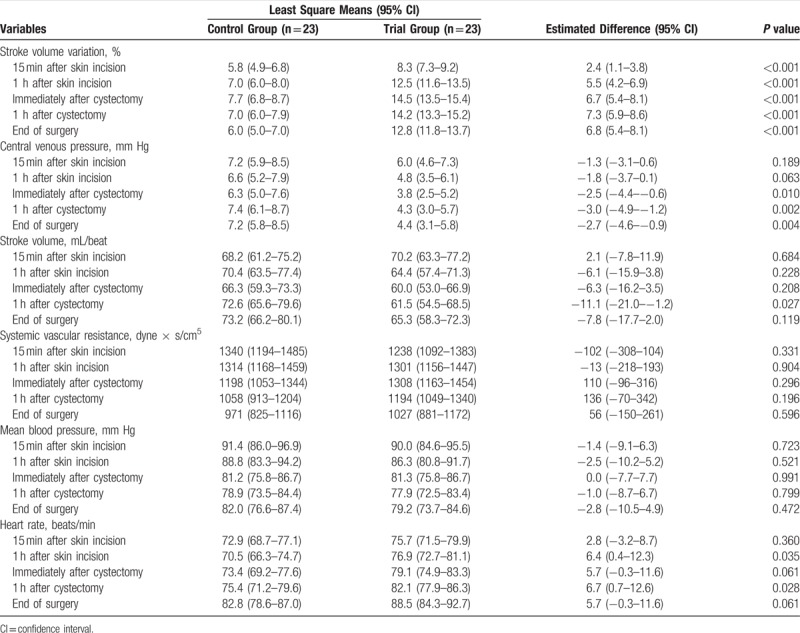
Intraoperative Hemodynamic Variables.

**Table 5:**
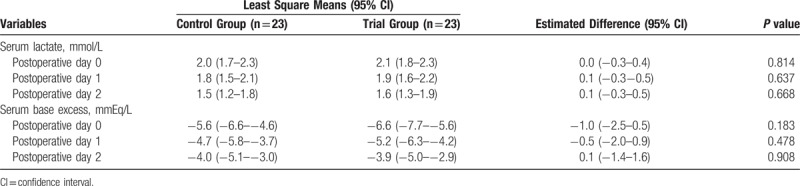
Postoperative Lactate and Base Excess Values.
